# Prophylactic cranial irradiation for limited‐stage small‐cell lung cancer in the magnetic resonance imaging era

**DOI:** 10.1002/cam4.5082

**Published:** 2022-07-27

**Authors:** Lihua Pan, Xingwen Fan, Lifang Wang, Yihua Wang, Yaqi Li, Yingshan Cui, Hong Zheng, Qiong Yi, Kailiang Wu

**Affiliations:** ^1^ Department of Radiation Oncology Fudan University Shanghai Cancer Center Shanghai China; ^2^ Department of Oncology Affiliated Hospital of Jining Medical University Jining Shandong China; ^3^ Department of Oncology, Shanghai Medical College Fudan University Shanghai China; ^4^ Shanghai Key Laboratory of Radiation Oncology Shanghai China

**Keywords:** brain metastasis, cranial irradiation, magnetic resonance imaging, small cell lung cancer, survival

## Abstract

**Background:**

We investigated the role of prophylactic cranial irradiation (PCI) in limited‐stage small‐cell lung cancer (LS‐SCLC) according to tumor response in the magnetic resonance imaging (MRI) era.

**Methods:**

We retrospectively evaluated patients with LS‐SCLC without brain metastases (BMs) on MRI who achieved either complete response (CR) or partial response (PR) after initial chemoradiotherapy at our center from 2006 to 2017.

**Results:**

This study comprised 116 patients (median age, 58 years; men, 92; women, 24). After initial chemoradiotherapy, 53 patients achieved CR, while 63 patients achieved PR. Eighty‐three patients received PCI. Patients who received PCI had better overall survival (OS, 5‐year: 52.5% vs. 35.1%; *p* = 0.012) and progression‐free survival (PFS, 5‐year: 45.0% vs. 28.2%; *p* = 0.001) and a lower incidence of BMs (5‐year: 18.3% vs. 39.4%; *p* = 0.010). In the subgroup analysis, PCI improved OS (5‐year: 67.8% vs. 46.7%, *p* = 0.005) and PFS (5‐year: 65.2% vs. 35.0%, *p* = 0.021) and decreased BM risk (5‐year: 12.1% vs. 52.4%, *p* = 0.002) for patients with CR. However, PCI had no benefit (5‐year OS: 40.5% vs. 35.6%, *p* = 0.763; 5‐year BMs: 24.6% vs. 31.9%, *p* = 0.561) for patients with PR.

**Conclusions:**

Tumor response remained an important factor for selecting patients for PCI in the MRI era. PCI should be recommended for patients with LS‐SCLC who achieve CR after initial thoracic chemoradiotherapy.

## Graphical abstract

Tumor response remains an important factor for selecting patients with limited‐stage small‐cell lung cancer for prophylactic cranial irradiation (PCI) in the magnetic resonance imaging era. PCI should still be considered for patients with complete response after initial thoracic radiotherapy.

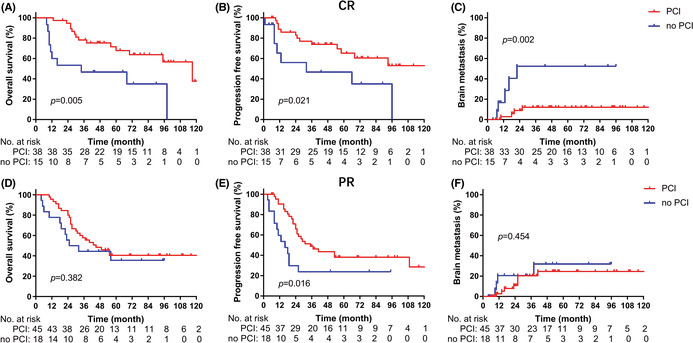

## 1 INTRODUCTION

Small‐cell lung cancer (SCLC) represents 13%–15% of all lung cancer cases, and limited‐stage SCLC (LS‐SCLC) comprises one‐third of SCLCs at the time of diagnosis.^1^ More than 50% of patients with SCLC develop brain metastases (BMs), and approximately 45% of those who achieve complete response (CR) to initial therapy develop BMs as the only relapse.^2^ Prophylactic cranial irradiation (PCI) can reduce the incidence of BMs by almost 50%. However, improvement in overall survival (OS) was not confirmed until a meta‐analysis, published in 1999, which assessed seven randomized trials from 1977 to 1995, reported a 5.4% increase in 3‐year OS in the entire PCI group.^3^ Based on this meta‐analysis, PCI is recommended for patients with LS‐SCLC who achieve CR after initial therapy.

In the magnetic resonance imaging (MRI) era, the beneficial role of PCI in SCLC has been challenged. However, nearly 16% of patients with unsuspected BMs missed by computed tomography (CT) are identified by MRI.^4^ Clearly, these patients may benefit more from PCI treatment. Nevertheless, more asymptomatic BMs were identified by MRI during follow‐up, and salvage whole‐brain radiation or stereotactic radiosurgery (SRS) was still effective.^5^ Since Takahashi et al.^6^ published their study on the negative effect of PCI on extensive‐stage SCLC, an increasing number of studies have questioned the role of PCI in LS‐SCLC.^7^, ^8^, ^9^, ^10^

Another major problem is that the scope of application of PCI in LS‐SCLC has expanded gradually without high‐level evidence. In a meta‐analysis,^3^ only patients with CR after initial treatment were recruited. However, PCI was recommended, in clinical trials and guidelines, for patients with major response,^11^, ^12^ response,^13^, ^14^ or even no progression^15^, ^16^ after chemoradiotherapy. We hypothesized that only patients with CR after initial treatment could benefit from PCI, as tumor cells from uncontrolled chest lesions are likely to spread to the brain after PCI and form new BMs.

## 2 METHODS

### 2.1 Ethics statement

All procedures performed in studies involving human participants were in accordance with the ethical standards of the institutional and/or national research committee and with the 1964 Declaration of Helsinki and its later amendments or comparable ethical standards. The study design was approved by the Institutional Review Board of Fudan University Shanghai Cancer Center, Shanghai, China. The requirement for written informed consent was waived owing to the retrospective nature of the study. The authors are accountable for all aspects of the work in ensuring that questions related to the accuracy or integrity of any part of the work are appropriately investigated and resolved.

### 2.2 Patient selection

We conducted a retrospective review of patients treated for LS‐SCLC at our center from 2006 to 2017. All personal data were deidentified. Patients were included if they achieved partial response (PR) or CR after initial thoracic chemoradiotherapy and underwent MRI before and after treatment. Patients were excluded if they underwent surgical resection, had Stage I SLC, were lost to follow‐up, or developed BMs <1 month after the last chemotherapy.

### 2.3 Treatment

Patients with LS‐SCLC usually receive one or two cycles of induction chemotherapy, accompanied by concurrent chemoradiotherapy. Etoposide and cisplatin (EP) with conventional fractionated radiotherapy at a total dose of 60 Gy in 30 fractions or 55 Gy in 22 fractions was the most used regimen at our center. PCI was recommended for patients who achieved CR or PR. The decision for or against PCI was made by the patients. The PCI dose was 25 Gy delivered in 10 fractions. All patients received intensity‐modulated radiotherapy to the thorax, and most received two‐dimensional radiotherapy for PCI. The hippocampus was not avoided, and memantine was not prescribed.

### 2.4 Tumor response and survival outcomes

Tumor response was assessed using the Response Evaluation Criteria in Solid Tumors (version 1.1),^17^ at 4–6 weeks after the completion of chemoradiotherapy, using a CT scan of the chest with contrast, ultrasound of the abdomen and pelvis, and MRI of the brain with contrast. CR was defined as complete tumor regression, with the short diameter of lymph node smaller than 1 cm, and tumor markers returned to normal. PR was defined as tumor regression of more than 30%, but with tumor residual or abnormal tumor markers. Exploratory PR was divided into major and minor PR, according to whether regression was more than 70%. OS was defined as the interval between the start of chemotherapy and death from any cause or last follow‐up. Progression‐free survival (PFS) was defined as the time to disease progression or recurrence, including intracranial and extracranial recurrence, or death. Progression was identified clinically or radiographically. Pathological confirmation was not necessary.

### 2.5 Statistical analyses

We compared the treatment‐related characteristics between the two groups using Pearson's chi‐squared or Fisher's exact test. The median follow‐up was estimated using the reverse Kaplan–Meier method. All time‐to‐event analyses were conducted using the date of initiation of chemotherapy as the starting date. OS curves, PFS curves, and the cumulative incidence of BMs were estimated using the Kaplan–Meier method and compared using the log‐rank test. Patients who remained alive were censored. Uni‐ and multivariate analyses of OS and BMs were performed using the Cox proportional hazards regression model. Age, sex, and concurrent chemoradiotherapy were used to calculate propensity scores. Patients were paired 1:1 using logistic regression estimation with a nearest neighbor matching algorithm based on these factors. All statistical analyses were conducted using IBM SPSS Statistics for Windows (version 26.0) (IBM Corp.). A two‐sided *p* < 0.05 was considered statistically significant.

## 3 RESULTS

### 3.1 Patient and treatment characteristics

**FIGURE 1:**
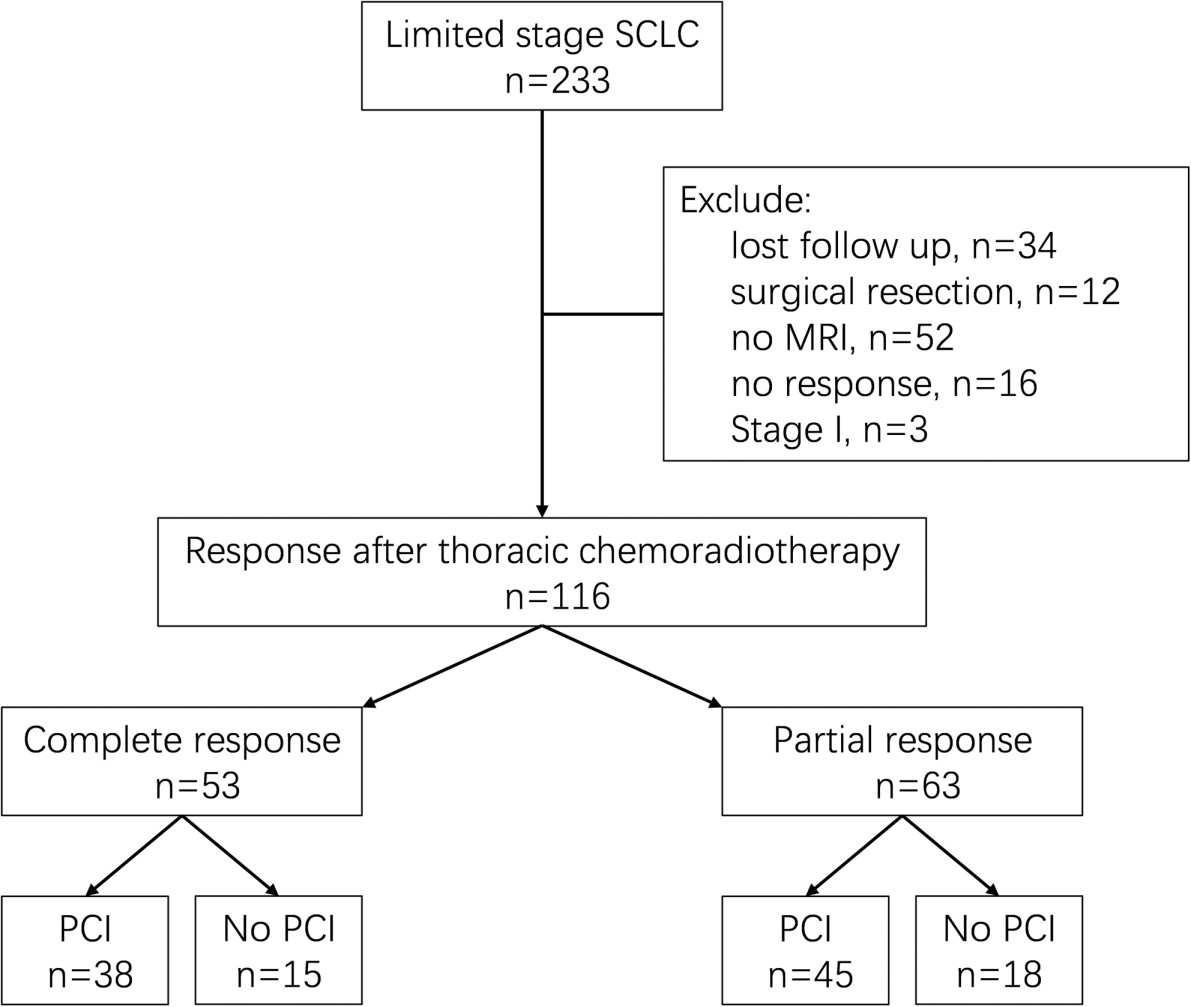
Flow diagram for patient selection. MRI, magnetic resonance imaging; PCI, prophylactic cranial irradiation; SCLC, small cell lung cancer.

A total of 233 patients with LS‐SCLC were treated at our center between January 2006 and December 2017, of whom 116 were included in the analysis. The flow diagram is shown in Figure 1, and the patient and treatment characteristics are shown in Table 1. The median age was 58 (range, 33–77) years. The proportion of male patients was 79.3%, and that of Stage III LS‐SCLC was 93.1%. Most patients (70.7%) received concurrent chemoradiotherapy. Fifty‐three (45.7%) patients achieved CR, while 63 (54.3%) patients achieved PR, including 50 (43.1%) patients with major PR, and 13 (11.2%) patients with minor PR. Eighty‐three (71.6%) patients received PCI, and the median time between thoracic radiation and PCI was 83 days. The baseline characteristics were balanced between the PCI and non‐PCI groups, except that more patients received concurrent chemoradiotherapy in the PCI group (81.9% vs. 42.4%; *p* < 0.0001).

**TABLE 1:** Patient and treatment characteristics

Characteristic	Total *N* (%)	PCI *N* (%)	Non‐PCI *N* (%)	*p*‐value
Age, years				
Median	58	57	61	0.28
≥60	54 (46.6)	36 (43.4)	18 (54.5)	
<60	62 (53.4)	47 (56.6)	15 (45.5)	
Sex				
Male	92 (79.3)	64 (77.1)	28 (84.8)	0.35
Female	24 (20.7)	19 (22.9)	5 (15.2)	
Family history				
Yes	19 (16.4)	13 (15.7)	6 (18.2)	0.74
No	97 (83.6)	70 (84.3)	27 (81.8)	
Active smokers				
Yes	76 (65.5)	51 (61.4)	25 (75.8)	0.14
No	40 (34.5)	32 (38.6)	8 (24.2)	
TNM stage				
II	8 (6.9)	6 (7.2)	2 (6.1)	0.82
III	108 (93.1)	77 (92.8)	31 (93.9)	
Chemotherapy drug				
EP	106 (91.4)	77 (92.8)	29 (87.9)	0.40
others	10 (9.6)	6 (7.2)	4 (12.1)	
Chemotherapy cycle				
≥5	75 (64.7)	55 (66.3)	20 (60.6)	0.57
<5	41 (35.3)	28 (33.7)	13 (39.4)	
Concurrent chemoradiotherapy				
Yes	82 (70.7)	68 (81.9)	14 (42.4)	<0.0001
No	34 (29.3)	15 (18.1)	19 (57.6)	
Radiotherapy fraction				
Conventional	82 (70.7)	57 (68.7)	25 (75.8)	0.45
Hyper‐	2 (1.7)	1 (1.2)	1 (3.0)	
Hypo‐	32 (27.6)	25 (30.1)	7 (21.2)	
Radiotherapy starting before 3 cycles of chemotherapy				
Yes	59 (50.9)	44 (53.0)	15 (45.5)	0.46
No	57 (49.1)	39 (47.0)	18 (54.5)	
Tumor response				
CR	53 (45.7)	38 (45.8)	15 (45.5)	0.97
PR	63 (54.3)	45 (54.2)	18 (54.5)	

Abbreviations: CR, complete response; EP, etoposide and cisplatin; PCI, prophylactic cranial irradiation; PR, partial response; TNM, tumor–node–metastasis.

### 3.2 Survival

As of June 2022, the median follow‐up was 81.0 (95% confidence interval [CI]: 67.2–94.8) months. The median OS and PFS in all patients were 60.0 (95% CI: 19.6–100.4) and 53.0 (95% CI: 23.7–82.3) months, respectively. The 2‐, 5‐, and 10‐year OS rates were 79.3%, 56.2%, and 33.1%, respectively. The equivalent PFS rates were 64.7%, 44.1%, and 31.3%, while the incidence of BMs was 20.6%, 23.5%, and 23.5%, respectively.

**FIGURE 2:**
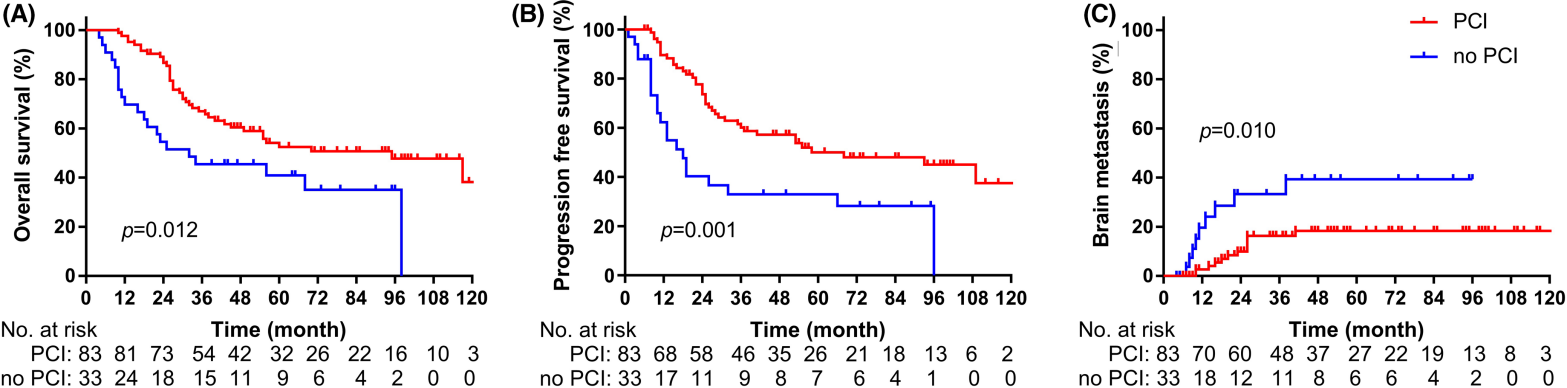
Survival between whether received PCI for all patients. (A) Overall survival. (B) Progression‐free survival. (C) Brain metastasis risk. PCI, prophylactic cranial irradiation.

Patients in the PCI group had better OS (median: 95.0 [95% CI: 51.9–138.1] months vs. 32.0 [95% CI: 0.0–66.8] months; 5‐year: 52.5% vs. 35.1%; *p* = 0.012; Figure 2A), better PFS (median: 68.0 [95% CI: 26.5–109.5] months vs. 18.0 [95% CI: 11.3–24.7]; 5‐year: 45.0% vs. 28.2%; *p* = 0.001; Figure 2B), and a lower incidence of BMs (5‐year: 18.3% vs. 39.4%; *p* = 0.010; Figure 2C).

In the multivariate analysis, younger age hazard ratio (HR: 0.56 [95% CI: 0.32–0.99], *p* = 0.045) and PCI (HR: 0.49 [95% CI: 0.27–0.89], *p* = 0.019) were associated with better OS (Table 2). Only PCI (HR: 0.33 [95% CI: 0.13–0.85], *p* = 0.022) was associated with lower brain metastasis (Table 2).

**TABLE 2:** Multivariate analysis of overall survival and brain metastases

Factor	Overall survival		Brain metastases	
	HR (95% CI)	*p*‐value	HR (95% CI)	*p*‐value
Age, years				
≥60	Reference	**0.045**	Reference	0.438
<60	0.56 (0.32–0.99)		1.47 (0.56–3.88)	
Sex				
Male	Reference	0.438	Reference	0.350
Female	0.69 (0.28–1.75)		0.51 (0.13–2.08)	
Family history				
No	Reference	0.613	Reference	0.161
Yes	1.18 (0.62–2.27)		1.91 (0.77–4.70)	
Active smokers				
No	Reference	0.758	Reference	0.203
Yes	1.13 (0.53–2.41)		0.46 (0.34–1.53)	
TNM stage				
II	Reference	0.364	Reference	0.542
III	1.67 (0.55–5.01)		0.65 (0.17–2.56)	
Chemo cycle				
≥5	Reference	0.735	Reference	0.803
<5	1.11 (0.61–2.04)		0.88 (0.32–2.41)	
Conventional fraction radio				
No	Reference	0.336	Reference	0.800
Yes	1.35 (0.73–2.51)		0.87 (0.29–2.60)	
Radio starting time				
Before 3rd chemo	Reference	0.067	Reference	0.074
No	1.72 (0.96–3.07)		2.60 (0.91–7.41)	
Tumor response				
CR	Reference	0.084	Reference	0.441
PR	1.67 (0.93–2.98)		1.45 (0.56–3.76)	
PCI				
No	Reference	0.019	Reference	**0.022**
Yes	0.49 (0.27–0.89)		0.33 (0.13–0.85)	

Abbreviations: Chemo, chemotherapy; CI, confidence interval; CR, complete response; HR, hazard ratio; PCI, prophylactic cranial irradiation; PR, partial response; Radio, radiotherapy; TNM, tumor–node–metastasis.

Bold indicates the values *p* < 0.05.

After propensity score matching, all the factors were balanced between the PCI and no‐PCI group, including whether patients received concurrent chemoradiotherapy (Table S1) and PCI improved OS and PFS, as well as decreasing BMs, the same as before propensity score matching (Figure S1).

### 3.3 Response effect

**FIGURE 3:**
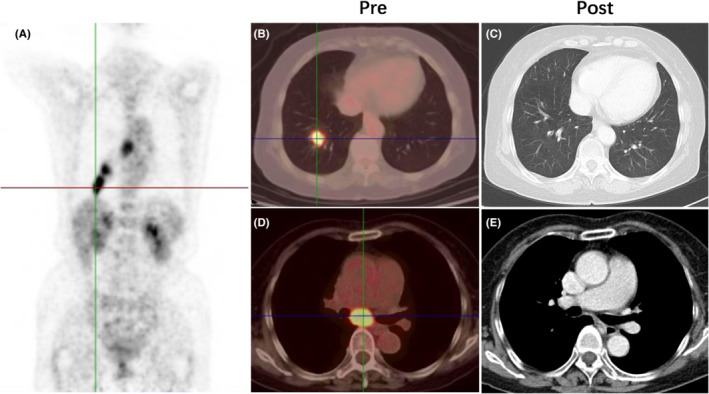
Typical case of patient with CR. (A) PET shows a tumor in the right lower lung and mediastinal lymph nodes before treatment. (B) Tumor in the right lower lung before treatment. (C) Tumor in the right lower lung disappeared after treatment. (D) Tumor in the subcarinal lymph node before treatment. (E) Tumor in the subcarinal lymph node regressed completely (diameter became smaller than 1 cm). CR, complete response; PET, Positron emission image.

A typical case of CR and major and minor PR are shown in Figure 3; Figures S2 and S3. Better early response after thoracic radiotherapy had better PFS (*p* = 0.0008, Figure S4B) and tended to have better OS (*p* = 0.143, Figure S4A). There was also no significant difference of BM between the two different responses (*p* = 0.622, Figure S4C).

**FIGURE 4:**
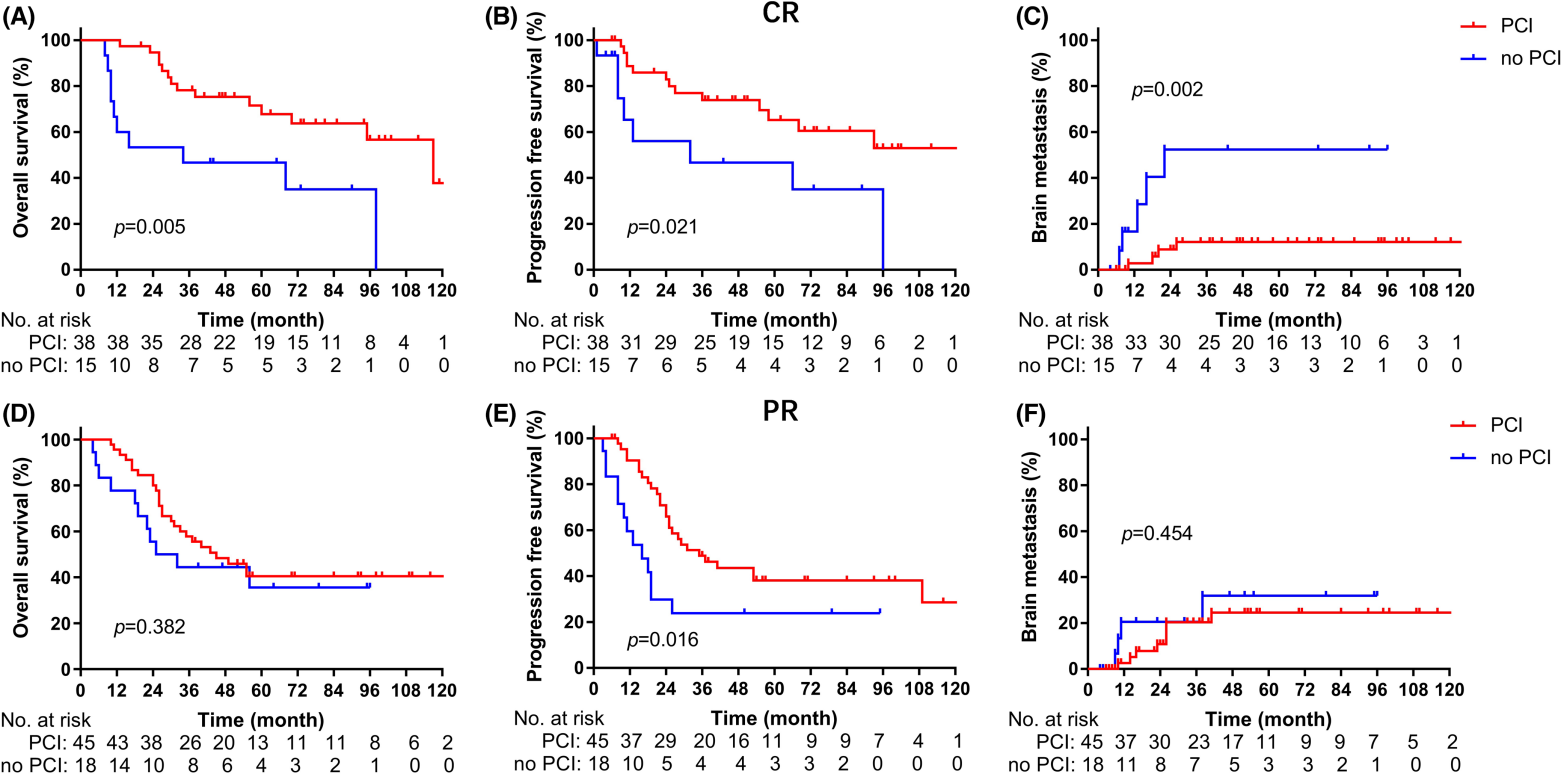
Survival between whether received PCI for patients with different responses. (A–C) patients with CR. (D, E) patients with PR. (A) Overall survival. (B) Progression‐free survival. (C) Brain metastasis risk. (D) Overall survival. (E) Progression‐free survival. (F) Brain metastasis risk. CR, complete response; PCI, prophylactic cranial irradiation; PR, partial response.

For patients with CR, the PCI group had better OS (5‐year: 67.8% vs. 46.7%, *p* = 0.005, Figure 4A), better PFS (5‐year: 65.2% vs. 35.0%, *p* = 0.021, Figure 4B), and lower BM (5‐year: 12.1% vs. 52.4%, *p* = 0.002, Figure 4C) than those in the no PCI group. However, for patients with PR, PCI neither improved OS (5‐year: 40.5% vs. 35.6%, *p* = 0.763, Figure 4D) nor decreased BM (5‐year: 24.6% vs. 31.9%, *p* = 0.561, Figure 4E). Even for patients with major PR, PCI still had no benefit (Figure S5).

## 4 DISCUSSION

The advantages of PCI for LS‐SCLC have been challenged in the MRI era, and several retrospective studies have been conducted to reassess its benefits. No OS benefit was observed in any of these studies,^7^, ^8^, ^9^, ^10^ while BM benefit was observed in some of them.^8^, ^10^ In this study, we retrospectively analyzed 116 patients with LS‐SCLC and MRIs who were treated in our center between 2006 and 2017 and found that PCI still benefitted those patients irrespective of their OS, PFS, or BM. Additionally, early response after thoracic radiotherapy was useful for selecting the right patients, and only patients from the CR subgroup benefitted from PCI in the MRI era.

In early exploration, nearly all studies selected patients with CR to assess the effect of PCI on LS‐SCLC.^2^, ^18^ However, the indication was expanded from CR to good response^11^, ^18^ and PR,^13^, ^14^ without high‐quality, evidence‐based data. This is because insufficient assessment methods in the 1970s and 1980s, such as chest radiography, may magnify the tumor response. CR evaluated by chest radiography contains some of the major responses.^18^ In our opinion, cancer cells would spread from extracranial tumors without sufficient control of the brain tissue and develop new metastases after PCI. Therefore, a high dose of PCI cannot reduce the incidence of BMs compared to the standard dose.^18^ In this study, the 5‐year BM rate in patients with CR who received PCI was only 12.1%, which was lower than that reported in other studies (29%–40%).^3^, ^18^ We believe that this was due to advances in imaging techniques, allowing more accurate CR to be selected. Several prospective, single‐arm, or randomized clinical trials (NCT04829708 and NCT04168281) have been conducted to assess the effect of PCI on LS‐SCLC.^19^, ^20^ Both trials enrolled patients with CR and PR after chemoradiotherapy. Preset subgroup analysis by CR and PR is suggested; otherwise, it is not possible to determine whether patients with CR still require PCI in the MRI era.

Since the publication of a Japanese study in 2017 that did not find a positive effect of PCI on extensive‐stage SCLC,^6^ a significant declining trend in PCI use in both extensive‐stage SCLC and LS‐SCLC has been observed.^21^ Furthermore, the neurotoxicity of PCI has been criticized. There are two verified methods to rescue radiation‐induced cognitive dysfunction: hippocampal avoidance and memantine administration.^22^, ^23^, ^24^ Many experimental strategies have also been explored, such as exercise,^25^ acupuncture,^26^, ^27^ and stem cell transplantation.^28^, ^29^ Additionally, salvage radiotherapy at the asymptomatic BM stage is effective.^30^, ^31^ Based on our findings, appropriate stratified treatment should be administered: PCI with hippocampal avoidance for patients with CR, and MRI surveillance and salvage radiotherapy (whole‐brain radiotherapy or SRS) for those with PR. We plan to initiate a prospective randomized controlled phase III study to test this idea. In this study, the 5‐year OS rates of patients with CR and major and minor PR were 61.9%, 44.3%, and 20.5%, while 5‐year PFS rates were 57.5%, 29.7%, and 9.7%, respectively. Therefore, early deep response, especially CR, is the surrogate endpoint to achieve long‐term survival. Two strategies were explored to improve CR after initial treatment: The first was to increase the radiation dose. Unfortunately, nearly all schemes failed, including conventional fractionation to 66 Gy,^15^ hypofractionated once daily to 65 Gy,^32^ and hyperfractionated twice daily to 60 Gy.^13^ The second strategy was to change the chemotherapy regimen. Given that the early response after the first cycle of chemotherapy is critical,^33^ determining whether to change chemotherapy drugs for those who do not achieve PR after the first cycle is useful. Concurrent EP plus induction of irinotecan and cisplatin,^34^ or consolidation of irinotecan and cisplatin,^35^ seemed to improve CR (by approximately 40%); however, this changing drug model failed in a randomized phase III study (JCOG 0202).^36^ Because both drugs act on similar targets (DNA topoisomerase I/II), patients resistant to etoposide are highly likely to be resistant to irinotecan. Studies on paclitaxel or other anticancer drugs, especially sensitive drugs selected using the organoid method,^37^, ^38^, ^39^ are warranted to explore the role of changing drugs in patients who are not sensitive to the first cycle of EP in the future. Additionally, the introduction of immune checkpoint blockade offers prolonged benefits in a select subset of patients.^40^

There are several limitations to this study. Notably, this was a retrospective study, and it was difficult to avoid inherent bias, such as more patients with concurrent chemoradiotherapy having received PCI. Although we used propensity score matching to balance this bias, a prospective randomized trial is still needed to verify our results in the future. Second, the time to assess the response was not uniform; the median time was 4 (range: 0–13) weeks, and only 40.5% of patients underwent tumor marker testing. Further, it was sometimes difficult to distinguish CR from major PR by CT; positron emission computed tomography (PET/CT) could supply more information. Additionally, not all the patients (61.2%) received regular follow‐ups, and the salvage treatment data after BM were incomplete.

## 5 CONCLUSION

PCI still benefitted patients with LS‐SCLC in the MRI era. Early tumor response after initial treatment was important for selecting patients suitable for PCI. Patients with CR assessed by CT, but not PR, showed improved OS and decreased BM by PCI.

## AUTHOR CONTRIBUTIONS

Lihua Pan: Data Curation, Formal Analysis, and Writing—Original Draft; Xingwen Fan: Conceptualization, Data Curation, Formal Analysis, Funding Acquisition, and Writing—Review and Editing; Lifang Wang: Investigation, Resources, and Validation; Yihua Wang: Data Curation, and Project Administration; Yaqi Li: Data Curation, and Software; Yingshan Cui: Data Curation; Hong Zheng: Data Curation; Qiong Yi: Data Curation; Kailiang Wu: Conceptualization, Funding Acquisition, and Writing—Review and Editing.

## FUNDING INFORMATION

This work was supported by the National Natural Science Foundation of China (grant numbers: 81872551 and 81903252) and the Key Clinical Specialty Project of Shanghai.

## CONFLICT OF INTEREST

The authors have no conflicts of interest to declare.

## Supporting information


Table S1
Figure S1
Figure S2
Figure S3
Figure S4
Figure S5

## Data Availability

Research data are stored in an institutional repository and will be shared upon request to the corresponding author.
