# Supplementary material for: Prophylactic cranial irradiation for limited‐stage small‐cell lung cancer in the magnetic resonance imaging era
Source: Cancer Med. 2022 Jul 27;12(3):2484–92. doi: 10.1002/cam4.5082 (PMC9939136; doi:10.1002/cam4.5082)
Supplement: Supplementary file 1 — Table S1 Figure S1 Figure S2 Figure S3 Figure S4 Figure S5 [file CAM4-12-2484-s001.docx]

Table S1. Patient and treatment characteristics after propensity score matching.

| Characteristic | Total  N (%) | PCI  N (%) | Non-PCI  N (%) | *P*-value |
| --- | --- | --- | --- | --- |
| Age, years  Median  ≥60  <60 | 59  29 (50.0)  29 (50.0) | 57  13 (44.8)  16 (55.2) | 61  16 (55.2)  13 (44.8) | 0.431 |
| Sex  Male  Female | 46 (79.3)  12 (20.7) | 21 (72.4)  8 (27.6) | 25 (86.2)  4 (13.8) | 0.195 |
| Family history  Yes  No | 10 (17.2)  48 (82.8) | 5 (17.2)  24 (82.8) | 5 (17.2)  24 (82.8) | 1.000 |
| Smoking history  Yes  No | 41 (70.7)  17 (29.3) | 19 (65.5)  10 (34.5) | 22 (75.9)  7 (24.1) | 0.387 |
| TNM stage  II  III | 3 (5.2)  55 (94.8) | 2 (6.9)  27 (93.1) | 1 (3.4)  28 (96.6) | 0.553 |
| Chemotherapy drug  EP  others | 52 (89.7)  6 (10.3) | 26 (89.7)  3 (10.3) | 26 (89.7)  3 (89.7) | 1.000 |
| Chemotherapy cycle  ≥ 5  < 5 | 36 (62.1)  22 (37.9) | 18 (62.1)  11 (37.9) | 18 (62.1)  11 (37.0) | 1.000 |
| Concurrent chemoradiotherapy  Yes  No | 28 (48.3)  30 (51.7) | 14 (48.3)  15 (51.7) | 14 (48.3)  15 (51.7) | 1.000 |
| Radiotherapy fraction  Conventional  Hyper-  Hypo- | 35 (60.3)  1 (1.7)  22 (37.9) | 14 (48.3)  0 (0.0)  15 (51.7) | 21 (72.4)  1 (3.4)  7 (24.1) | 0.138 |
| Radiotherapy starting before 3 cycles of chemotherapy  Yes  No | 26 (44.8)  32 (55.2) | 13 (44.8)  16 (55.2) | 13 (44.8)  16 (55.2) | 1.000 |
| Tumor response  CR  PR | 26 (44.8)  32 (55.2) | 11 (37.9)  18 (62.1) | 15 (51.7)  14 (48.3) | 0.291 |

Abbreviations: CR, complete response; PCI, prophylactic cranial irradiation; PR, partial response; TNM, Tumor–Node–Metastasis.


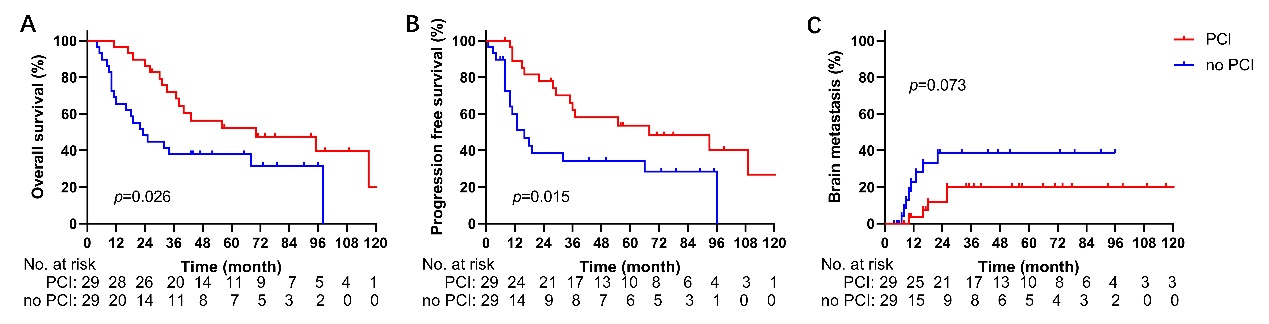
Figure S1. Survival after propensity score matching. A, overall survival. B, progression free survival. C, brain metastasis risk. PCI, prophylactic cranial irradiation.


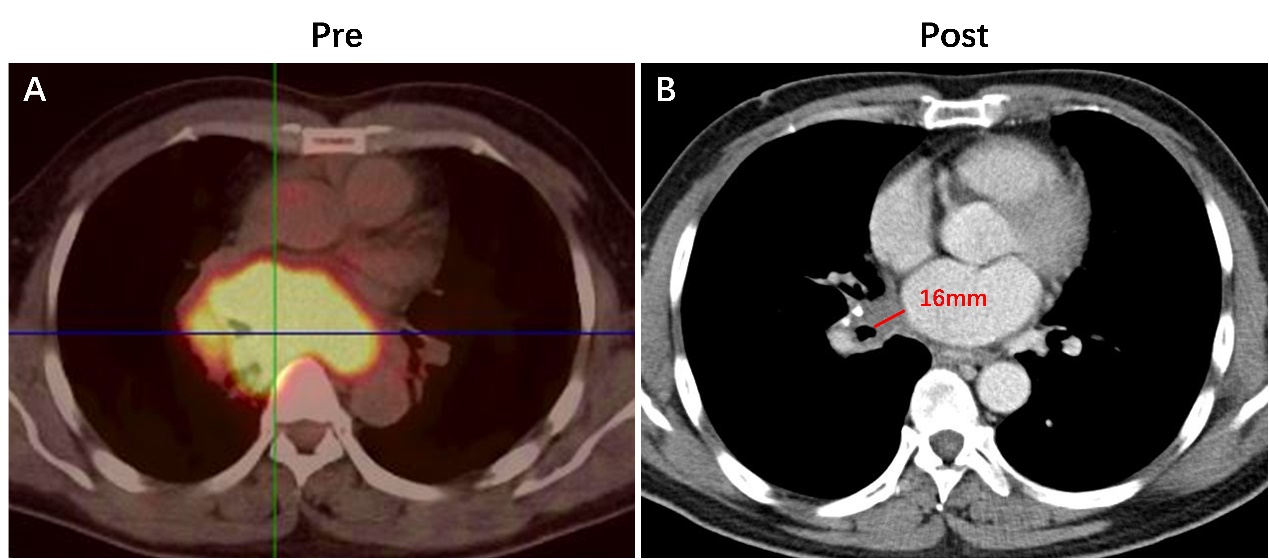


Figure S2. Typical case for major partial response. A, tumor with high metabolism in the hilar before treatment. B, tumor regressed significantly but the short diameter greater than 1cm after treatment.


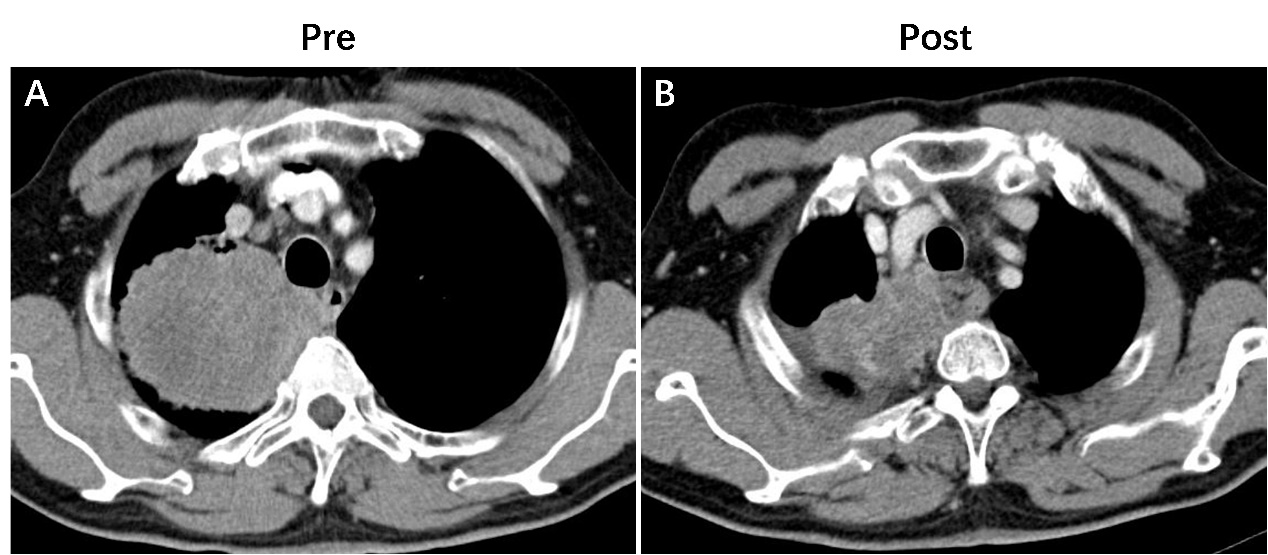


Figure S3. Typical case for minor partial response. A, big tumor of paramediastinum before treatment. B, tumor regression more than 30% but less than 70% after treatment.


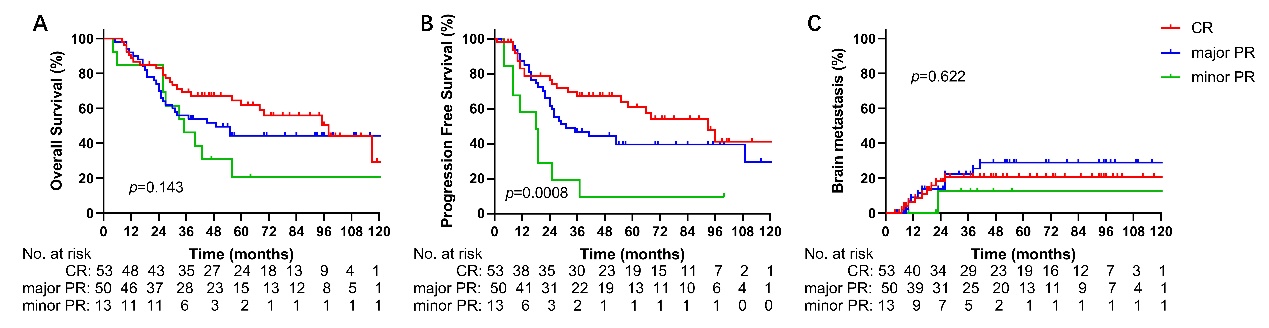


Figure S4. Survival between different response. A, overall survival. B, progression free survival. C, brain metastasis risk. CR, complete response; PR, partial response.


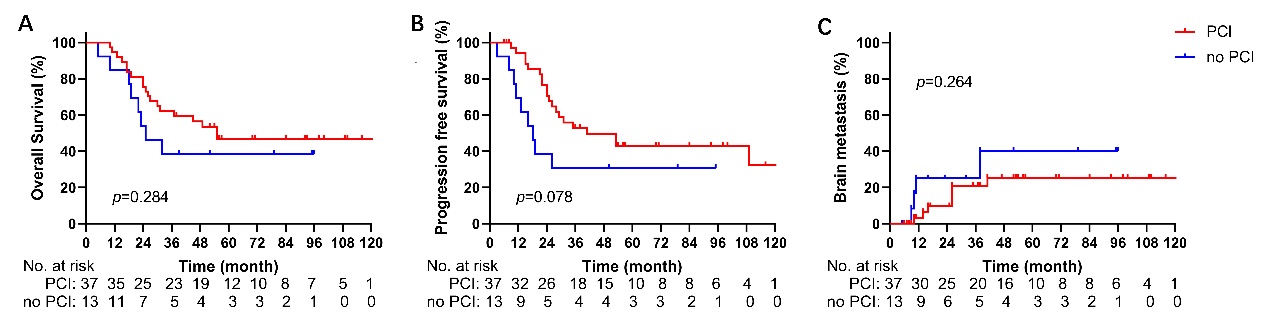


Figure S5. Survival for patients with major partial response. A, overall survival. B, progression free survival. C, brain metastasis risk. PCI, prophylactic cranial irradiation.
